# RNA editing and alternative splicing of the insect nAChR subunit alpha6 transcript: evolutionary conservation, divergence and regulation

**DOI:** 10.1186/1471-2148-7-98

**Published:** 2007-06-27

**Authors:** Yongfeng Jin, Nan Tian, Jun Cao, Jing Liang, Zhaolin Yang, Jianning Lv

**Affiliations:** 1 Institute of Biochemistry, College of Life Sciences, Zhejiang University(Zijingang Campus), Hangzhou, Zhejiang, ZJ310058, P.R. of China; 2 Institute of Biochemistry, Zhejiang Sci-Tech University, Hangzhou, Zhejiang, ZJ 310018, P.R. China

## Abstract

**Background:**

RNA editing and alternative splicing play an important role in expanding protein diversity and this is well illustrated in studies of nicotinic acetylcholine receptors (nAChRs).

**Results:**

Here, we compare the RNA editing and alternative splicing of the nAChR alpha6 subunit genes from different insects spanning ~300 million years of evolution– *Drosophila melanogaster, Anopheles gambiae, Bombyx mori, Tribolium castaneum *and *Apis mellifera*. The conserved and species-specific A-to-I RNA editing occurred across all species except *A. gambiae*, which displayed extraordinarily short flanking intronic sequences. Interestingly, some A-to-I editing sites were a genomically encoded G in other species. A combination of the experimental data and computational analysis of orthologous alpha6 genes from different species indicated that RNA editing and alternative splicing predated at least the radiation of insect orders spanning ~300 million years of evolution; however, they might have been lost in some species during subsequent evolution. The occurrence of alternative splicing was found to be regulated in distinct modes and, in some cases, even correlated with RNA editing.

**Conclusion:**

On the basis of comparative analysis of orthologous nAChR alpha6 genes from different insects spanning ~300 million years of evolution, we have documented the existence, evolutionary conservation and divergence, and also regulation of RNA editing and alternative splicing. Phylogenetic analysis of RNA editing and alternative splicing, which can create a multitude of functionally distinct protein isoforms, might have a crucial role in the evolution of complex organisms beyond nucleotide and protein sequences.

## Background

RNA editing is a process that results in the synthesis of proteins that are not directly encoded in the genome. One type of RNA editing involves the modification of individual adenosine bases to inosine in RNA by ADAR enzymes (adenosine deaminases acting on RNA) [1,2]. Because inosine acts as guanosine during translation, A-to-I conversion in coding sequences leads to amino acid changes and often entails changes in protein function [2-4]. A-to-I RNA editing is common in animals and is associated with various neurological functions [3,4]. *Caenorhabditis elegans, Drosophila melanogaster *and *Mus musculus *mutants lacking ADAR enzymes display predominantly distinct neurological phenotypes [5-8]. In addition to amino acid changes, the editing and subsequent destabilization of the RNA duplex present in the 5' or 3'-untranslated regions (UTRs) could alter the stability, transport or translation of the mRNA [2,9]. Moreover, RNA editing may influence alternative splicing decisions [10].

Alternative splicing is a major contributor to transcriptomic and proteomic complexity, disease, and development. Alternative splicing may affect the protein sequence in two ways: (i) by deleting or inserting a sequence and creating long and short isoforms, or (ii) by substituting one segment of the amino acid sequence for another [11]. An indication for the first pathway is that truncated isoforms often act as dominant-negative regulators of the full-length isoform's activities [12,13]. In contrast, the second mode is capable of creating, from mutually exclusive alternative sequences, a multitude of functionally distinct protein isoforms and thus might have a crucial role in the evolution of complex organisms [11]. As both RNA editing and alternative splicing can lead to the inclusion of alternative amino acid sequences into proteins, functionally distinct isoforms are likely to be generated [14]. Therefore, editing and alternative splicing provide a powerful posttranscriptional means for fine-tuning of gene expression at the cellular and organismal levels.

Nicotinic acetylcholine receptors (nAChRs) mediate the fast actions of the neurotransmitter acetylcholine (ACh) in both vertebrates and invertebrates [15]. An extraordinary feature of the insect nAChR genes is that they can potentially create many different mRNAs by RNA editing and alternative splicing. More than 30,000 alpha6 nAChR isoforms are theoretically possible through RNA editing and alternative splicing, without considering any linkage between these events [16]. The alternatively spliced exons are organized into two clusters. The exon 3 and 8 clusters contain 2 and 3 alternative versions, respectively [16]. Seven adenosines could be modified in *D. melanogaster *alpha6, four of which are also edited in the alpha6 ortholog in the tobacco budworm *Heliothis virescens*. However, although these RNA A-to-I editing sites are conserved between *D. melanogaster *and *H. virescens*, they are not shared with the equivalent nAChR subunit of *Anopheles*, which is considered to be an example of convergent evolution [17]. It is possible that different alpha6 isoforms may interact with distinct sets of receptor guidance cues. RNA editing and alternative splicing of the nAChR alpha6 pre-mRNA may therefore be central to the mechanisms specifying transmitter affinity, channel conductance and ion selectivity.

The recently sequenced genomes of 12 *Drosophila *species [18], the mosquito *A. gambiae *[19], the silkworm *B. mori *[20], the honeybee *A. mellifera *[21], and *T. castaneum *[18] have renewed interest in molecular and functional diversity in the insect nAChR alpha6 gene. Recent analysis reveals bees and wasps (Hymenoptera) are at the base of the radiation of Holometabolous insects [22,23]. Here, we compare the RNA editing and alternative splicing of the nAChR alpha6 gene from these insects spanning ~300 million years of evolution. These sequence comparisons provide insight into the evolution of the nAChR alpha6 gene and indicate that many isoforms have arisen by RNA editing and alternative splicing events. These findings also suggest that expressing a diverse nAChR alpha6 repertoire is more important than the actual sequence of each isoform. In this article, we describe the A-to-I RNA editing and alternative splicing found in insect nAChR alpha6 genes, as well as their evolutionary conservation and divergence, and regulation. In addition, we provide an example of a strong correlation between RNA editing and alternative splicing.

## Results

### Comparison of the nAChR subunit alpha6 genes from different insect species

To obtain insight into the functional diversity, the regulation of expression, and the evolution of nAChR alpha6, we have compared the sequence of the nAChR alpha6 genes in *Drosophila *to other species. The organisms analyzed consisted of 13 Dipteran species, including 12 *Drosophila *species (*D. melanogaster*, *D. simulans, D. sechellia, D. yakuba, D. erecta*, *D. ananassae*, *D. pseudoobscura*, *D. persimilis*, *D. willistoni*, *D. mojavensis*, *D. virilis *and *D. grimshawi*) and one mosquito (*A. gambiae*), the Lepidopteran *B. mori *(silkworm), the Coleopteran *T. castaneum *(red flour beetle) and the Hymenopteran *A. mellifera *(honeybee). The sequences from these species allowed us to analyze the evolution of the nAChR alpha6 gene over at least 300 million years and across phylogenetic orders. The overall organization of the nAChR alpha6 genes of these insect species is quite similar, but there are a few subtle differences. The nAChR alpha6 genes possess two versions for exon 3 in most species, while no such tandem duplication of coding exon 3 can be found in the *A. mellifera *genome. Although nAChR alpha6 genes have three versions for exon 8 in most species, only two alternatives for the equivalent exon are observed in the *A. gambiae *and *B. mori *genomes.

**Figure 1:**
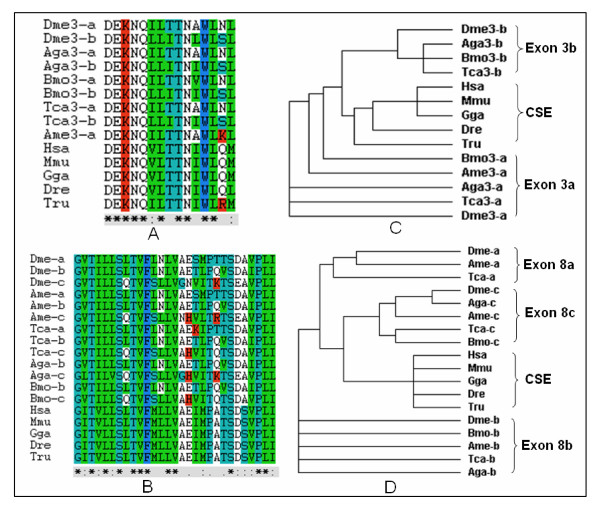
Multiple alignments and phylogenetic analysis of duplicated exon nucleotide sequences. (A, B) Multiple alignments of amino acid sequences of duplicated exon 3 (A) and exon 8 (B) sequences and their counterparts from orthologs in other species, respectively. The alternative exons are labeled 'a' and 'b' or 'c'. (C, D) Cladogram of insect duplicated exon DNA sequences and vertebrate orthologous constitutive exons corresponding to the alignments shown in (A, B). For each cluster, the amino acid sequences of each alternative exon from each species were aligned using the Clustal W program and phylogenetic trees generated. The branches contained the vertebrate constitutive exons (CSE), invertebrate alternative exons 8a or exons 3a, alternative exons 8b or exons 3b and alternative exons 8c, respectively. Abbreviations: Hsa, *H. sapiens *(NM_000746.3); Dre, *D. rerio *(NP_957513.1); Mmu, *M. musculus *(NP_031416.2); Gga, *G. gallus *(NP_989512.1); Tru, *T. rubripes *(CAG03274.1); Dme, *D. melanogaster *(CG4128) [16]; Aga, *A. gambiae *[17]; Bmo, *B. mori *(CH379590); Tca, *T. castaneum *(CM000280); Ame, *A. mellifera *[21].

We next analyzed the evolutionary relationship of the alternative exons from *D. melanogaster*, *A. gambiae, B. mori, T. castaneum *and *A. mellifera *nAChR alpha6 genes. For these analyses we used *D. melanogaster *as the representative *Drosophila *species. Phylogenetic analyses indicated that *A. mellifera *did not contain an ortholog to the second alternative exon 3 while *B. mori *and *A. gambiae *lacked an ortholog to the first alternative exon 8[17,24] (Figure 1). The orthologs to the second alternative exon 8 were very highly conserved, with all amino acid sequences identical (Figure 1), although they are not highly conserved at the nucleotide level. The hierarchy of amino acid conservation of the alternative exons 8 of nAChR subunit alpha6 genes was exon 8b > exon 8a > exon 8c. Phylogenetic analysis of the protein products of equivalent duplicated exons showed that members of a duplicated pair were more similar to each other than to the exons from other genes (Figure 1). This evidence suggests that exon duplication predated at least the radiation of insect orders spanning ~300 million years of evolution.

We next analyzed the evolutionary relationship of the alternative exons within the *Drosophila *species. All three exon 8 variants had orthologs in each species. All three exon 8 orthologs were very highly conserved in these species, and were identical at the amino acid level. Surprisingly, the orthologs to the alternative exon 8b were identical even at the nucleotide level. We determined a similar hierarchy of nucleotide conservation of the alternative exons 8 of nAChR subunit alpha6 genes within the *Drosophila *species, namely exon 8b > exon 8a > exon 8c.

### Conservation and divergence of alternative splicing

**Figure 2:**
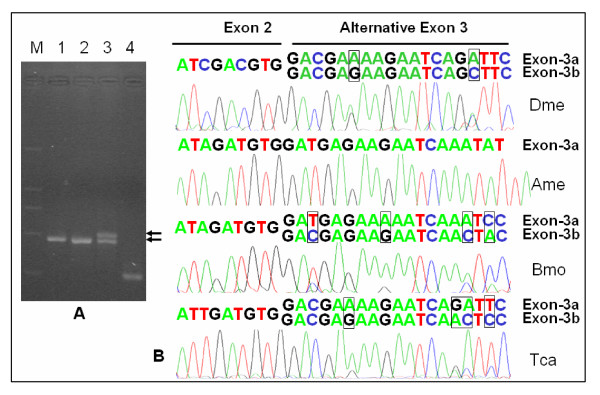
Species-specific alternative splicing patterns. (A) Analysis of species-specific alternative splicing patterns using an RT-PCR-based strategy. 1: *D. melanogaster*; 2: *A. mellifera; *3:*B. mori*; 4: *T. castaneum*. Primers used for amplification of splice products were DmDa-5-1 and DmDa-3-1 for *D. melanogaster*, AmDa-5-3 and AmDa-3-1 for *A. mellifera*, BmDa-5-4 and BmDa-3-1 for *B. mori*, TcDa-5-1 and TcDa-3-1 for *T. castaneum*, respectively (Table 1). The migration positions of PCR products corresponding to transcripts with one or two alternative duplicated exon variants are indicated. Because the sequence between the specific primers in *T. castaneum *is smaller than in other species, its band is smaller. (B) Comparison of the boundary sequences of sites in exon 2 and alternative exons 3 among nAChR alpha6 orthologs of *D. melanogaster *(Dme), *B. mori *(Bmo), *T. castaneum *(Tca) and *A. mellifera *(Ame). Direct sequencing of these RT-PCR products (A) confirmed that these duplicated exons are alternatively spliced. Different nucleotides in the alternative exons 3a and 3b (in box) showed a mixed signal.

We were interested in understanding the alternative splicing of the nAChR alpha6 transcripts and in particular whether this is regulated. We first analyzed how the alternative exons 3 were regulated. The vast majority of tandemly duplicated exons (99.4%) are likely to be involved in mutually exclusive alternative splicing events [14]. The Reverse Transcription Polymerase Chain Reaction (RT-PCR) showed a very clear band in *D. melanogaster *and *T. castaneum *adult cDNA, as in *A. mellifera *with constitutive exon 3 (Figure 2A). Direct sequencing of these amplification products confirmed that these duplicated exons were alternatively spliced (Figure 2B). Sequence analysis of 30 cDNAs also showed that no duplicated exons were spliced together. These results indicated that the vast majority of exons were likely to be involved in mutually exclusive alternative splicing, which was consistent with EST and cDNA data analysis, although a duplicate of exon 3 was even found in cDNA [16].

**Figure 3:**
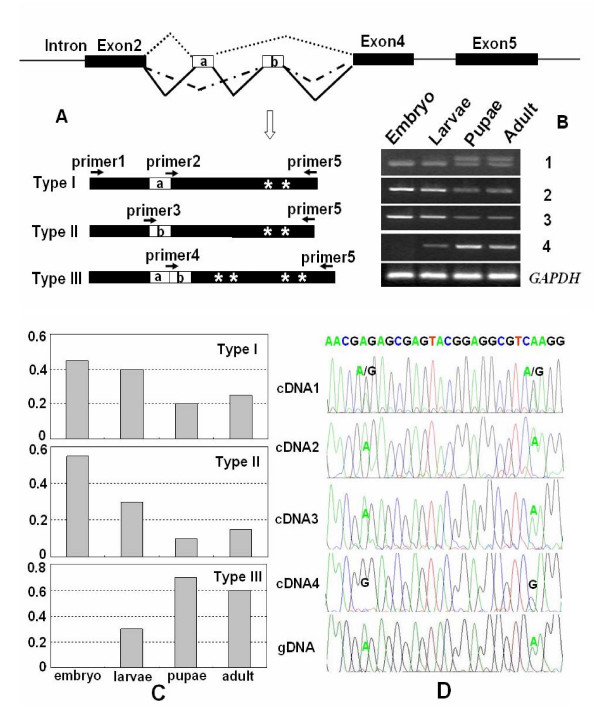
Editing of *B. mori *nAChR alpha6 splice forms (A) The structure and alternative splicing patterns of the alpha6 gene in the regions surrounding the alternative exons 3. Three main variants, depending on the alternative exon 3 versions: type I (alternative exon 3a), type II (alternative exon 3b), type III (alternative exons 3a and 3b). Boxes represent exons and the line represents introns. The black boxes represent constitutive exons and the open boxes represent exons that are duplicated in tandem. "*" represents the editing sites. The migration positions of PCR products corresponding to transcripts with one or two alternative duplicated exon variants are indicated with arrows. Primer1, 2, 3, 4, 5 refer to BmDa-5-4, BmDa-5-8, BmDa-5-9, BmDa-5-10, and BmDa-3-1, respectively (Table 1). (B) Analysis of alternative splicing in silkworm embryo, larvae, pupae, and adult using RT-PCR. 1, 2, 3, 4 indicated splice forms amplified using forward primer (primer1, or 2, or 3, or 4), and reverse primer (primer5), respectively. (C) Frequency of splice forms in silkworm embryo, larvae, pupae, and adult. RNA was isolated from each developmental stage and used for RT-PCR. The RT-PCR products were cloned and analyzed. 20 cDNA clones were sequenced for every stage. (D) Comparison of the editing levels (A/G signal) of sites in exon 4 among distinct splice forms. cDNA 1, 2, 3, 4 indicated splice forms in Figure 3B.

The RT-PCR showed a distinct pattern in *B. mori *(Figure 2A), not seen in *D. melanogaster *and *T. castaneum*. Total silkworm RNA harvested from silkworms at various stages of development was used as a template for RT-PCR with primers surrounding the exon 3 (Figure 3A). At the embryo stage, only one band could be amplified, and direct sequencing indicated a mixed sequence signal of two nucleotides, confirming the existence of alternative splicing of exon 3. The existence of alternative splicing in the embryonic transcripts was further confirmed by independent sequencing of cloned cDNAs of RT-PCR products. This band, which resulted from type I and type II alternative splicing, decreased slightly during development, exhibiting a very low level in the pupal and adult stages (Figure 3B). However, another slightly larger band appeared during development, displaying a very low level in the larvae but increasing rapidly during the stages from pupa to adult (Figure 3B). Sequencing of cloned cDNAs of this product indicated the existence of splice type III alternative splicing, which included both alternative exons, and retained the opening frame of the spliced RNA.

To elucidate how the alternative splicing patterns of exon 3 were developmentally regulated, we designed the specific primers based on three alternative variant cDNAs (Figure 3A). Consequently, the expression of splice type I and II appeared to be tightly regulated in a similar manner, exhibiting an abundant level in the embryo, and decreasing slightly during the pupal stage to the adult level. In contrast, the expression of splice type III showed a distinct mode during development, exhibiting a very low level in the embryo, and increasing rapidly during the pupal stage to the adult level (Figure 3B). The expression analysis using RT-PCR was consistent with the results derived from RT-PCR clones (Figure 3C). Thus, the alternative splicing patterns of exon 3 in silkworm were different from the other insect species.

**Figure 4:**
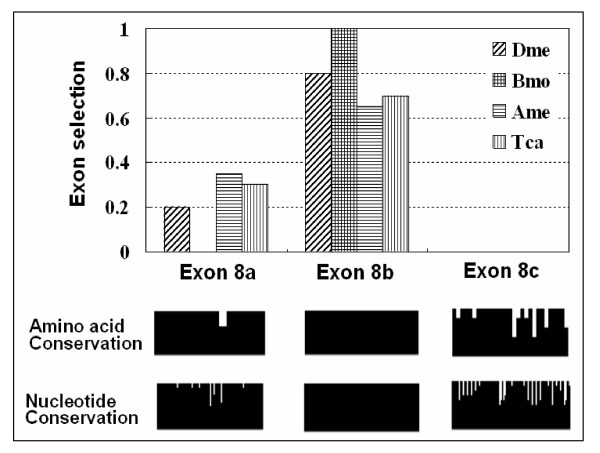
A similar hierarchy of selection efficiency of alternative exons 8 of nAChR subunit alpha6 genes among *D. melanogaster *(Dme), *B. mori *(Bmo)*, T. castaneum *(Tca) and *A. mellifera *(Ame): exon 8b > exon 8a > exon 8c. Expression of alternative exons 8 is identical with its conservation. The diagram indicates amino acid conservation from multiple alignments of duplicated exon 8 sequences from orthologs in different species. 20–30 cDNA clones were sequenced for each species. The diagram also indicates nucleotide conservation from multiple alignments of duplicated exon 8 sequences from orthologs within *Drosophila *species.

Next, to determine how the alternative exons 8 were regulated developmentally, we first performed RT-PCR on RNA isolated from silkworm embryos, larvae, pupae and adults by use of primers flanking exon 8. The identity of the PCR product was confirmed by cloning and sequencing. Sequence analysis of transcripts, derived from different staged RNA independent amplification experiments, indicated that only cDNAs containing exon 8b were included, but no cDNA containing exon 8c was included. This suggested that only exon 8b was spliced while exon 8c expression was very low in these developmental stages. We next tested whether the selection pattern of exon 8 alternative splicing observed in silkworm was conserved in *D. melanogaster*, *T. castaneum *and *A. mellifera*, where nAChR subunit alpha6 has three versions for exon 8. Interestingly, RT-PCR analysis revealed an identical hierarchy of selection efficiency among *D. melanogaster*,*T. castaneum *and *A. mellifera*: exon 8b > exon 8a > exon 8c (Figure 4). Considering the identical trend between selection efficiency and conservation of alternative exons 8, it is proposed that expression of alternative exons 8 might be closely related with its conservation (Figure 4). This phenomenon might reflect evolutionary trajectories and/or differential functional constraints.

### Evolutionary conservation and divergence of nAChR alpha6 RNA editing

**Figure 5:**
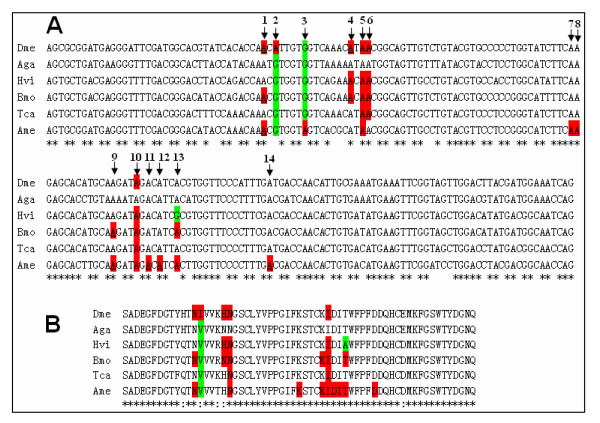
The conserved and species-specific A-to-I RNA editing. Alignment of the homologous exon 5 genomic nucleotide (A) and amino acid (B) sequences of nAChR subunit alpha6 genes from *D. melanogaster *(Dme), *A. gambiae *(Aga), *H. virescens *(Hvi)*, B. mori *(Bmo), *T. castaneum *(Tca) and *A. mellifera *(Ame). RNA editing of the nAChR subunit alpha6 genes from *D. melanogaster*, *H. virescens *and *A. gambiae *have been previously described [16, 17]. The editing sites from positions 1–14 (A) and amino acids (B) are shaded in red. Those sites constitutively G (A) and amino acids (B) are shaded in green at the editing sites. RNA editing sites, which were not evidently detected as a mixed sequence signal G and A, but revealed by sequencing of cDNA clones, are underlined.

Similar to substitution alternative splicing, RNA editing causes amino acid changes by substituting individual nucleotides. Although RNA A-to-I editing occurred in *Drosophila *transcripts, it was not present in the equivalent nAChR subunit of *Anopheles *[16,17]. To determine whether orthologous nAChR subunits were also RNA-edited in other insect species, we have subsequently analyzed the nAChR alpha6 genes from *B. mori, T. castaneum *and *A. mellifera*. Several sites were either determined as pure G signals, or as mixed sequence signals of G and A, while the nucleotide in the reference genomic DNA was A at these positions. We also checked for genetic variation in these alpha6 regions, using PCR amplification on silkworm genomic DNA, applying primers surrounding the equivalent regions and direct sequencing. As a result, it was revealed that the nucleotide in genomic DNA was adenosine at these positions, confirming that post-transcriptional modifications occur in this case. We have identified a total of ten A-to-I RNA editing sites within silkworm alpha6 transcripts, seven of which are located in exon 5 (Figure 5A). Sequence analysis of transcripts showed that six amino acids could be changed by seven possible A-to-G transitions in exon 5 (Figure 5B).

**Figure 6:**
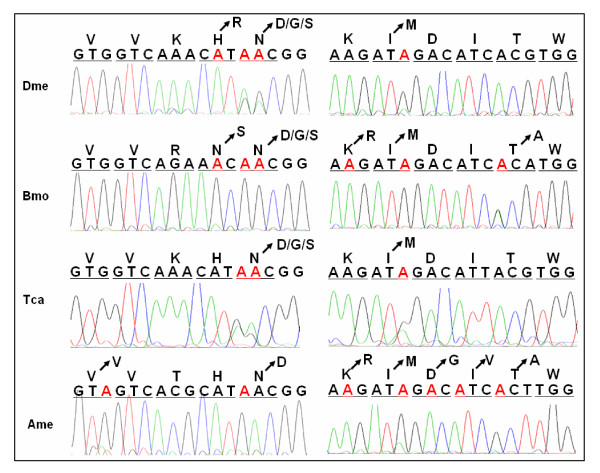
Comparison of the editing levels among four orders. The editing sites (in red) and their editing levels (A/G signal) of sites 3–6 and 9–13 among the nAChR alpha6 orthologs from *D. melanogaster *(Dme), *B. mori *(Bmo)*, T. castaneum *(Tca) and *A. mellifera *(Ame) are shown. RNA editing of the nAChR subunit alpha6 genes from *D. melanogaster *have been previously described [16]. Some RNA editing sites were not evidently detected as a mixed sequence signal G and A, but revealed by sequencing of cDNA clones.

We have subsequently analyzed the nAChR alpha6 genes from *T. castaneum *and *A. mellifera*. We demonstrated that editing also occurred in both the alpha6 homolog of *T. castaneum *and *A. mellifera*, with a quite different pattern of editing (Figure 5, 6). These sites were originally discovered through sequence analysis of cDNAs that were subsequently compared with genomic DNA. In each case, A was observed in the genomic sequence with G at the corresponding position in numerous cDNAs. A-to-I RNA editing occurred across the species except *A. gambiae *with its extraordinarily short flanking intronic sequences (Figure 5, 6). There are up to 11 A-to-I RNA editing sites in *A. mellifera*. During the preparation of this paper, six A-to-I RNA editing sites were found in *A. mellifera *[24], which were identical with 6 of the 11 RNA editing sites in our experiment. The editing sites 5 and 10 in nAChR alpha6 were conserved among the four orders of insect, represented by *D. melanogaster*, *B. mori*, *T. castaneum *and *A. mellifera *(Figure 5). The RT-PCR clone data revealed that editing was detectable at site 1 in *B. mori *and *A. mellifera*, as in *Drosophila*, albeit at a very low level. Site 4 was edited at low levels in *D. melanogaster*, but almost completely edited in *B. mori*, and undetectable in *T. castaneum *and *A. mellifera*. Interestingly, site 3 was specifically edited in *A. mellifera *but it constituted a genomically encoded G in other species. Sites 7 and 8 were markedly edited in *A. mellifera*, while editing was undetectable in other species. In contrast, site 6 was edited in *D. melanogaster*, *B. mori *and *T. castaneum*, but undetectable in *A. mellifera *(Figure 5). Overall, editing sites and levels of the alpha6 homolog differed among species, possessing conserved and species-specific editing sites in each species. However, this was the most highly conserved RNA editing event yet reported in invertebrates.

### Some A-to-I editing sites were a genomically encoded G in other species

The nAChR subunit alpha6 genes were subject to RNA editing in *D. melanogaster*, *B. mori, T. castaneum *and *A. mellifera*. Interestingly, there were several examples in which some A-to-I editing sites were a genomically encoded G in some species. For example, the alpha6 site 13 was edited in *B. mori *and *A. mellifera*, while the site 13 in the alpha6 ortholog α*7–2 *in the tobacco budworm *H. virescens *(Lepidoptera) was a genomically encoded G (Figure 5A). The site 3 was edited in *A. mellifera*, while the homologous sites in *A. gambiae, H. virescens*, *B. mori *and *T. castaneum *were a genomically encoded G (Figure 5A). Similarly, the alpha6 site 2 was edited in *Drosophila *[16]; however, the alpha6 homologous sites in other species were a genomically encoded G (Figure 5A). Although we did not know how general this phenomenon was, this led us to consider the possibility that RNA editing might act as an evolutionary intermediate form between single nucleotide polymorphism (SNP) sites, maintaining partial conservation at the protein and functional level despite sequence divergence at the DNA level.

### Correlation between RNA editing and alternative splicing

The vast majority of tandemly duplicated exons (99.4%) are likely to be involved in mutually exclusive alternative splicing events [14]. The alternative splicing patterns of the duplicated exon 3 were conserved in the chosen insect species, except the silkworm. It remained to be determined whether the alternative splicing pattern of mutually exclusive exons was changed because the signal ensuring the splicing of pairs of alternative exons was disturbed in the silkworm. RT-PCR and direct sequencing of the cDNAs derived from adult transcripts showed that two A-to-G substitutions occurred in exon 4 of the silkworm nAChR subunit alpha6 gene (Figure 3D). However, these two A-to-G substitutions were undetectable in embryonic transcripts. Interestingly, sequence analysis of 80 cDNA clones derived from embryonic, larvae, pupae and adult transcripts, respectively, revealed two A-to-G substitutions in exon 4 of the silkworm in splice type III, but not in splice types I and II. PCR amplification of silkworm genomic DNA and direct sequencing revealed that adenosine was the nucleotide in the genomic DNA at both positions, confirming that A-to-I editing occurred in this case. These results suggest that specific editing in exon 4 and splice type III of exon 3 might be closely related.

To determine whether coordinated RNA editing and alternative splicing were conserved in other insects, we subsequently analyzed the nAChR alpha6 genes from *D. melanogaster*, *T. castaneum *and *A. mellifera*. In contrast to the silkworm, sequence analysis indicated that no A-to-G substitutions took place in exon 4 in these species. These results suggest that RNA-editing sites in exon 4 are species-specific in *B. mori*, which correlates with the species-specific alternative splicing pattern of the duplicated exon 3. To discern whether alternative splicing also correlates with RNA editing in other distant exons in the silkworm, we analyzed the A-to-I RNA editing sites in exon 5 of the nAChR subunit alpha6 gene. However, no evidence indicated any relation between alternative splicing patterns of exon 3 and editing in distant exon 5, suggesting that the alternative splicing patterns of exon 3 was not regulated by RNA editing in distant exon 5.

## Discussion

### Evolutionary implications

**Figure 7:**
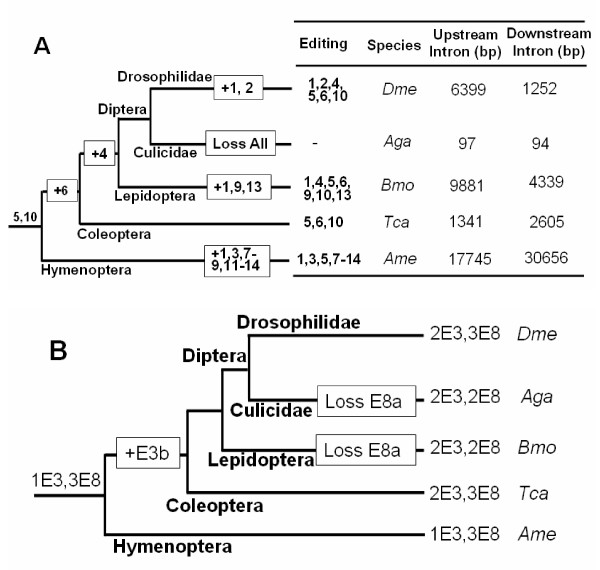
Phylogeny of RNA editing and duplicated exons of nAChR subunit alpha6 gene from *D. melanogaster *(Dme), *A. gambiae *(Aga), *B. mori *(Bmo), *T. castaneum *(Tca) and *A. mellifera *(Ame). RNA editing and duplicated exons of the nAChR subunit alpha6 genes from *D. melanogaster *and *A. gambiae *have been previously described [16,17]. Recent work suggests that the Hymenoptera are basal to the Coleoptera in the Endopterygota [22,23]. (A) Phylogeny of RNA editing and comparative sequence analysis of the flanking intronic sequences. Dashes indicate no detectable editing. Boxes denoting generations (+) and disappearances of particular editing events are indicated by boxes. The editing sites correspond to the number in Figure 5. (B) Phylogeny of duplicated exons. Boxes indicate generations (+) and disappearances of particular duplicated exons during evolution.

Our results indicate a high level of editing events in insect species spanning ~300 million years of evolution. Interestingly, RT-PCR analysis showed that RNA editing was absent in *A. gambiae*, indicating a divergence in molecules targeted by RNA editing within the Diptera order [17]. Interestingly, enzyme assays measuring conversion of adenosine to inosine in salivary gland homogenates of several mosquito species detected adenosine deaminase activity in *Culex quinquefasciatus *and *Aedes aegypti*, but not in *A. gambiae *[17]. However, Syt I is also edited in mosquitoes, sharing two editing sites with *Drosophila *species, and one mosquito-specific site [25]. This suggests that adenosine deaminase functions in the *Anopheles *lineage, as in *Drosophila*. A comparative sequence analysis of these species showed that the exon 5 was highly conserved at both the nucleotide and amino acid level among these species (Figure 5), but the flanking intronic sequences were highly divergent. The downstream intron 5 was extraordinarily short in length, 97 bp, in *A. gambiae*, while it was > 1 kb in *Drosophila*, > 4 kb in *B. mori*, > 2 kb *T. castaneum*, and > 30 kb in *A. mellifera*, respectively (Figure 7A). Likewise, the upstream intron 4 was also extraordinarily short, 94 bp, in *A. gambiae*, while it was much more than 1 kb in the other insect species (Figure 7A). The absence of editing in *Anopheles *correlated with the lack of downstream intronic sequences, which were necessary to direct editing by forming duplex RNA substrates for ADARs within larger, energetically stable RNA secondary structures. Similarly, sequential decrease and loss of editing in *A. gambiae*, through weakening of the ECS-editing site interaction to form poorer duplex RNA substrates for ADARs, could titrate in the edited form of the protein to the least advantageous level, or even an undetectable level.

RNA editing conserved between the orders Diptera and Lepidoptera for one nAChR gene was previously considered an example of convergent evolution [17]. However, our phylogenetic analysis of RNA editing in orthologous nAChR alpha6 genes from different species revealed a divergent evolution from a common ancestor. Moreover, this implies that divergent evolution from a common ancestor would have been accompanied by editing loss or gain in paralogous genes. We suggest that the data presented here comprise a credible phylogeny of RNA editing for a gene, graphically illustrating descent with modification (Figure 7A). RNA editing in insect nAChR subunit alpha6 genes predates at least the radiation of the Coleopteranand Hymenopteran orders, beginning with sites 5 and 10. New editing sites were probably generated and ancestral editing sites were lost in subsequent evolution through global intronic variation (Figure 7A). Our evidence suggests that *Anopheles *lost editing in the nAChR alpha6 gene during the evolution of the Diptera; such a loss might be consistent with the phylogenic evolution of the introns. The nAChR alpha6 genes possess tandem duplication of coding exons in their genomic sequences in insect species, which represents alternative spliced exons. Dating exon duplications through a combination of the available experimental data on alternative splicing in orthologous genes from different species and computational analysis indicated that the exon 3 and 8 duplications predated at least the radiation of insect orders spanning ~300 million years of evolution. Our results disproved the previous hypothesis that a duplication event gave rise to exon 8b and 8c before the divergence of an ancestor of *Drosophila *and *Anopheles*, whereas after the divergence a further duplication gave rise to an extra exon (exon 8a) in the *Drosophila *lineage [17]. However, our evidence suggests the possibility that divergent evolution from a common ancestor was accompanied by exon loss and generation of paralogous genes (Figure 7B). In the nAChR alpha6 gene, our evidence suggests that recent loss of copies of duplicated exons has occurred (e.g. exon 8a in *A. gambiae*). Moreover, divergent evolution from a common ancestor would have been accompanied by change in the alternative splicing pattern of mutually exclusive exons in paralogous genes (e.g. exon 3 in *B. mori*)

### RNA editing and SNP sites

The nAChR subunits alpha6 are subject to RNA editing in insect species. Interestingly, there were several examples where some A-to-I editing sites were a genomically encoded G in closely- related species. Although we do not know how general this interesting phenomenon is, this led us to consider the possibility that mRNA editing might act to maintain similarity at the protein and functional level despite sequence divergence at the DNA level. In plant mitochondria, mRNA editing might act to maintain similarity at the protein level [26]. Some genetic restoration events in plants and animals are proposed to be the result of a template-directed process that makes use of an ancestral RNA-sequence cache [27,28]. Therefore, the edited RNA-sequence might be taken as a template to synthesize DNA, thus causing changes at the DNA level. In addition, the most prevalent changes of substitutional RNA editing in the nucleus of higher eukaryotes are hydrolytic deaminations where a genomically encoded C or A is converted to U and I, respectively [29] Interesting, A/G and C/T(U) substitution were much more prevalent than other forms of SNP. Given these relations between RNA editing and SNP, it is pertinent to ask whether RNA editing might be an evolutionary predecessor to genomically fixed SNPs.

### Regulation of mutually exclusive alternative splicing

RNA editing and alternative splicing play an important role in expanding protein diversity and are commonly employed to enlarge the proteome. Since both processes may require conserved exonic and intronic elements, RNA editing may influence alternative splicing decisions or vice versa. There are a few examples of an association between alternative splicing and editing [30-34]. As RNA editing usually occurs close to exon/intron boundaries, this is likely to be a general phenomenon and suggests an important and novel role for RNA editing [30]. Moreover, the ADAR2 protein regulates its own synthesis by creating an alternative splice site that leads to an out-of-frame product [30,34]. The auto-editing of ADAR2 intron 4 by the ADAR2 adenosine deaminase is tightly coupled to splicing, as the modification of the dinucleotide AA to AI creates a new 3' splice site [34]. The editing site and the affected splice site are usually in close proximity to one another, and so RNA editing affected alternative splicing by creating or deleting splice sites [30-34]. Only one example indicates that the editing efficiency of a *Drosophila *gene correlates with a distant splice site selection where alternative splicing occurs downstream of editing. In contrast, no correlation is seen when editing occurs downstream of alternative splicing [35]. However, the result remains to be determined without considering the fact that RNA editing and alternative splicing are not regulated by similar developmental patterns.

In this study, RNA editing could affect the alternative splicing pattern by another mechanism because no new splice sites were generated or deleted. The vast majority of tandemly duplicated exons (99.4%) are likely to be involved in mutually exclusive alternative splicing events [14], therefore, mechanisms must exist to ensure that the splicing of pairs of alternative exons is strictly mutually exclusive, involved in competing base-pairing interactions [36], the steric hindrance of snRNP binding [37] and the dual spliceosome mechanisms [14]. If the signal involved in these mechanisms was disturbed, the alternative splicing pattern of mutually exclusive exons might be changed. Taken together, a model can be proposed to explain how editing and alternative splicing of pre-mRNA is coordinated. RNA editing in exon 4 might disrupt a splicing enhancer signal within exon 4, which can prevent the exon 3 cluster from splicing together. The disrupted splice site is now more efficient at splicing out the shorter intron, leading to the longer product. It is not exactly clear how an enhancer within this exon would alter the choice of a distant acceptor site, but there are previous studies showing that longer introns tend to be flanked by stronger splice sites [38]. To test whether A-to-I editing disrupts exon splicing enhancer (ESE) elements, we analyzed edited and unedited exon sequences with an ESE-finder program [39]. A-to-I editing in two sites was predicted by the ESE-finder to destroy the SF2/ASF (GGAACGA) and SRp40 (CGTCAAG) ESE motifs, respectively. Taken together, our results suggest that ESE disruption is the underlying mechanism of A-to-I editing that results in the change of the alternative splicing pattern. Conversely, if RNA editing does not occur, for example, in the silkworm embryo and in *D. melanogaster *and *T. castaneum*, mutually exclusive alternative splicing of the duplicated exons has arisen in the majority of transcripts. Alternatively, A-to-I editing might disrupt the other splice signals within exon 4 in the silkworm nAChR subunit alpha6, which controls mutually exclusive splicing of the duplicated exons. One protein has recently been identified that prevents all of the duplicated exon variants from being spliced together, which demonstrates that the duplicated exon variants are in fact capable of being spliced together but protein factors exist that repress this reaction [34]. A-to-I editing possibly relieves the repression on the upstream alternative duplicated exons, and as a result, the duplicated exon variants might be spliced together.

## Conclusion

We have documented the existence, evolutionary conservation, and regulation of RNA editing and alternative splicing in nAChR alpha6 from five insects spanning ~300 million years of evolution– *D. melanogaster, A. gambiae, B. mori, T. castaneum *and *A. mellifera*. A combination of the experimental data and computational analysis of orthologous alpha6 genes from different species indicated that RNA editing and alternative splicing predated at least the radiation of insect orders spanning ~300 million years of evolution; however, they might be lost in some species during subsequent evolution. The occurrence of alternative splicing was found to be developmentally regulated, and even correlated with RNA editing in some cases. Interestingly, some A-to-I editing sites represented a genomically encoded G in other species. Phylogenetic analysis of RNA editing and alternative splicing, which are capable of creating the multitude of functionally distinct protein isoforms, might have a crucial role in the evolution of complex organisms beyond nucleotide and protein sequences.

## Methods

### Materials

Total RNA was isolated from different developmental stages of *D. melanogaster, B. mori *(Qingsong X Haoyue), *A. mellifera *(ligustica Spinola) and *T. castaneum *(the red flour beetle) using the RNeasy Mini Kit (Qiagen, Germany) according to the manufacturer's protocol. Genomic DNA was isolated using the Universal Genomic DNA Extraction Kit (TaKaRa). RNA was stored at -80°C and genomic DNA was stored at 4°C. Plasmid DNA was purified using Qiagen plasmid isolation kit.

### Gene assemblies and analysis

The sequences of the nAChR subunit alpha6 genes from *D. melanogaster *and *A. gambiae *have been previously described [16,17]. The sequences of the nAChR subunit alpha6 genes for the other *Drosophila *species(*D. simulans, D. sechellia, D. yakuba, D. erecta*, *D. ananassae*, *D. pseudoobscura*, *D. persimilis*, *D. willistoni*, *D. mojavensis*, *D. virilis *and *D. grimshawi*), the silkworm *B. mori*, the honeybee *A. mellifera *and the red flour beetle *T. castaneum *were assembled from individual raw sequence reads available from the NCBI trace archives. Vertebrate alpha6 orthologs in *Homo sapiens *(human),*Danio rerio *(zebrafish), *Mus musculus *(mouse),*Gallus gallus *(chicken) and *Takifugu rubripes *(pufferfish) were identified by BLAST searches using the sequence of the most closely related organisms. The intron 7 sequence of the silkworm nAChR subunit alpha6 gene was determined using PCR and sequencing.

### Generation of full-length cDNA

Full-length cDNA clones were obtained using the 5'/3' RACE cDNA synthesis kit. First strand cDNA was synthesized from total RNA (0.5 μg) isolated from silkworm using SuperScript II reverse transcriptase primed with the oligo(dT)12–18 anchor primer according to the manufacturer's instructions (Invitrogen). The 5' and 3' RACE specific primers were designed according to the DNA sequence (Table 1). The DNA products were purified by using the Qiagen PCR purification kit and cloned into the pGEM-T Easy vector (Promega) according to the manufacturer's instructions. Isolation of recombinant clones was carried out using standard procedures.

**Table 1:** Primers used for the RT-PCR and PCR analysis

*Species*	*Experiment*	*Primer name*	*Primer sequence(5'-3')*
B. mori	5' RACE	BmDa-3-14	caccttaggttgtagtcattcca
	5' RACE	BmDa-3-13	tgttcggtgttatccttaagtcct
	Editing, AS	BmDa-5-1	gtgctgacgagggttttgacggga
	Editing, AS	BmDa-5-4	acgaaaaacgtctgctgaacgccct
	Editing, AS	BmDa-5-8	tggctaaacttggaatg
	Editing, AS	BmDa-5-9	tcatagatgtggacgagaag
	Editing, AS	BmDa-5-10	gctaaacttggacgag
	Editing, AS	BmDa-5-11	gtagcgcactgcccgtgtcca
	Editing, AS	BmDa-5-19	cgatgtagctgcttacgattgggt
	Editing, AS	BmDa-3-1	ctgattgccatcatatgtccagct
	Editing, AS	BmDa-3-2	ccagctaccaaacttcatatcac
	Editing, AS	BmDa-3-3	tcactgcacgatgatatgcggc
	Editing, AS	BmDa-3-5	ccgagtgtcaatttctccccagaat
	Intron7	BmDa-5-6	aggaatgccgggcaaaaagaaca
		BmDa-3-18	ttgagtcctattaacaggcgaacaga
	GAPDH	BmGAP5-1	ctactgttcatgccacaactgct
	GAPDH	BmGAP3-1	tgtacttgatgagatcaatgact
D. melanogaster	Editing, AS	DmDa-5-1	agttcggactgacgctgcagcagat
	Editing, AS	DmDa-5-2	cggatgagggattcgatggcacgt
	Editing, AS	DmDa-3-1	gcaagtaccactcgccatttgttat
	Editing, AS	DmDa-3-2	gaggcgaccatgaacatgatgcaat
A. mellifera	Editing	AmDa-5-1	gtgcggatgagggtttcgacggga
	Editing, AS	AmDa-5-2:	gaatgggtggactacaacctccaat
	Editing, AS	AmDa-5-3	ggacgtcacgagaaacgtttgttg
	Editing	AmDa-3-1	ctggttgccgtcgtaggtccagga
	Editing, AS	AmDa-3-3	tgtacatgagaatgtctggcttcca
	Editing, AS	AmDa-3-5	ctgtccaccaccatagcggcga
T. castaneum	Editing, AS	TcDa-5-1	aggggccgcacgaaaagcggctact
	Editing, AS	TcDa-5-2	aacgactataatctcaaatggaacg
	Editing, AS	TcDa-5-3	gtgcggatgagggtttcgacg
	Editing, AS	TcDa-3-1	cctggttgccgtcataggtccagct
	Editing, AS	TcDa-3-2	cataagaacatcaggcttccaca
	Editing, AS	TcDa-3-4	gcctactgcacgattatgtgcgg
	Editing, AS	TcDa-3-5	ttgaggcatttcgtggatatcagc
	Editing, AS	TcDa-3-6	caagaccactgacgacgctaccat

AS: Alternative splicing

### Analysis of gene expression by RT-PCR

Silkworm total RNA was reverse transcribed using SuperScript II RT and the resulting single-stranded cDNA product was treated with DNase at 37°C for 30 min. PCR amplification was carried out using cDNA from 10 ng of total RNA template in each reaction. The gene-specific primers for PCR were designed according to the nAChR alpha6 genomic sequence. Each splice product was amplified separately from bulk cDNA using a single spliceform-specific primer and a shared primer. Primer1, 2, 3, 4, 5 refer to BmDa-5-4, BmDa-5-8, BmDa-5-9, BmDa-5-10, and BmDa-3-1, respectively (Table 1). Amplification conditions were 35 cycles of 94°C for 30 s, 55-65°C for 30 s and 72°C for 30 s, followed by one cycle of 72°C for 10 min. Silkworm glyceraldehyde-3-phosphate dehydrogenase gene (GAPDH) transcripts were amplified as an external control.

### Analysis of alternative splicing forms

Total RNA was reverse transcribed using SuperScript II RT and the resulting single-stranded cDNA product was treated with DNase at 37°C for 30 min. The gene specific primers for PCR were designed according to the nAChR alpha6 genomic sequence (Table 1). PCR amplification was carried out using cDNA from 10 ng of total RNA template in each reaction. The products of RT-PCR were purified and cloned into the pGEM-T Easy vector (Promega, USA) and transformed with a JM109 competent cell. Recombinant clones were identified by restriction enzymes and PCR. Sequencing of selected clones was done using automatic DNA sequencer. cDNA sequences were determined by amplifying portions of the gene and directly sequencing the PCR product.

### Analysis of RNA editing

Analysis of RNA editing was performed using total RNA as the template for RT-PCR. RT-PCR was performed with the primer pairs mentioned above for nAChR alpha6 genes in different species. RT-PCR amplicons were either directly sequenced after gel purification or subcloned and individual cDNA-bearing plasmid clones subjected to sequencing. Primers for the nAChR subunit alpha6 exon 5 were used to amplify genomic DNA from the same tissues used for RNA isolation. The genomic PCR amplification product was subjected to direct sequencing to demonstrate that genomic products give a pure A signal at editing sites, ruling out a polymorphism. For subcloning, RT-PCR splice products were cloned into the pGEM-T Easy vector. Relative A-to-G abundance was determined by sequencing individual clones with plasmids containing appropriately sized inserts.

### nAChR subunit alpha6 sequences

The GenBank accession numbers of the nAChR alpha6 subunit genes are as follows: alpha6 cDNAs of *B. mori *variants are EF127797, EF127798, EF127799; alpha6 cDNAs of *T. castaneum *variants are from EF127806 to EF127810; alpha6 cDNAs of *A. mellifera *variants are from EF127800 to EF127805.

## Abbreviations

nAChR – nicotinic acetylcholine receptor

ADAR – adenosine deaminase acting on RNA

UTRs – untranslated regions

Ach – acetylcholine

RT-PCR – reverse transcription-polymerase chain reaction

EST – expressed sequence tag

SNP – single nucleotide polymorphism

ECS – editing site complementary sequence

ESE – exon splicing enhancer

GAPDH – glyceraldehyde-3-phosphate dehydrogenase

CSE – constitutive exons

AS – alternative splicing

Dme – *Drosophila melanogaster*

Bmo – *Bombyx mori*

Tca – *Tribolium castaneum*

Ame – *Apis mellifera*

Aga – *Anopheles gambiae*

## Authors' contributions

Y.J conceived and designed the experiments. N.T, J.C, J.L and Z.Y performed the experimental analysis in *B. mori, D. melanogaster*, *A. mellifera *and *T. castaneum*, respectively. J. L performed the sequence analysis and RNA secondary structure predictions. Y.J, N.T and J.C co-wrote this paper. All authors read and approved the final manuscript.
